# Post-marketing safety assessment of Tyrvaya: A real-world pharmacovigilance analysis based on the FDA Adverse Event Reporting System

**DOI:** 10.1097/MD.0000000000045032

**Published:** 2025-10-17

**Authors:** Chang-Zhu He, Qin Qiu, Song-Jie Lu, Fu-Li Xue, Wei-Yu Wang, Yu He

**Affiliations:** a Chengdu University of Traditional Chinese Medicine, Chengdu, Sichuan, China; b Department of Ophthalmology, Chengdu First People’s Hospital/Chengdu Integrated TCM and Western Medicine Hospital, Chengdu, Sichuan, China.

**Keywords:** adverse events, disproportionality analysis, FAERS, Tyrvaya, varenicline nasal spray

## Abstract

A preservative-free intranasal formulation of varenicline (Tyrvaya) has been developed and approved in the United States for the treatment of the signs and symptoms of dry eye disease (DED). Given its growing use in the clinical treatment of DED, understanding its safety in real-world settings is essential. This study aims to further assess the safety concerns after the launch of Tyrvaya. This study analyzed all adverse events (AEs) reports from the FDA Adverse Event Reporting System database, in which Tyrvaya was identified as the primary suspected drug, starting from the fourth quarter of 2021, to evaluate its safety in clinical practice. To ensure the accuracy and reliability of the study, we employed 4 types of disproportionality analyses: the reporting odds ratio, proportional reporting ratio, multi-item gamma Poisson shrinker (MGPS), and Bayesian confidence propagation neural network. Additionally, the Weibull distribution was utilized to model the risk of AEs over time. A total of 2178 patients and 5189 reports associated with Tyrvaya administration were collected. Positive signal detection of Tyrvaya at the system organ class level included respiratory, thoracic, and mediastinal disorders, product issues, and eye disorders. At the preferred term level, the AEs listed on the drug label were confirmed, including sneezing, cough, throat irritation, and instillation-site (nasal) irritation. Furthermore, several adverse reactions not listed in the medication leaflet but deemed clinically significant were identified, including blurred vision, burning sensation, upper-airway cough syndrome, nasal ulcers, dysphonia, abnormal dreams, photophobia, and eye swelling. Additionally, we identified potential adverse reactions, including headache, insomnia, and vertigo. This study identified known AEs associated with Tyrvaya and uncovered new AE signals that had not been previously reported. These findings offer clinicians additional safety insights for the use of Tyrvaya in treating DED.

## 1. Introduction

Dry eye disease (DED), also referred to as keratoconjunctivitis sicca, is a multifactorial condition affecting 1 or more components of the tear film, it leads to persistent tear film instability, characterized by an imbalance in tear film homeostasis, which may result from tear deficiency or excessive tear evaporation.^[1]^ Symptoms of DED may be intermittent or chronic, chronic dry eye is distinguished by both persistent symptoms and ongoing damage to the ocular surface. Common symptoms reported by DED patients include grittiness, itching, a foreign body sensation, tearing, burning, visual fatigue, and dryness.^[2]^ According to the report of the Tear Film and Ocular Surface Society (TFOS) Dry Eye Workshop II (DEWS II), the global prevalence of DED is 29.5%, with a rate of 28.1% in females and 24.9% in males.^[3]^ An estimated 4.88 million individuals aged 50 and older in the United States have DED, and the prevalence among younger people aged 18 to 34 is projected to increase. Older adults face a higher risk of developing DED due to factors such as medication use, hormonal changes, and underlying health conditions.^[4]^

A preservative-free intranasal formulation of varenicline (Tyrvaya) was developed and approved in the United States in 2021 for the treatment of DED signs and symptoms.^[5]^ Varenicline is a water-soluble, small-molecule nicotinic acetylcholine receptor (nAChR) agonist that binds with high affinity and selectivity to several human neuronal nAChR subtypes, including α4β2, α4α6β2, α4β3, and α3α5β4, where it exhibits partial agonist activity, and α7, where it acts as a full agonist.^[6,7]^ Tyrvaya is speculated to influence the trigeminal nerve endings in the anterior nasal cavity, thereby activating the nasolacrimal reflex. This activation stimulates the lacrimal functional unit, which comprises the meibomian glands, lacrimal glands, and goblet cells responsible for secreting components of the tear film, including mucin, aqueous, and lipid layers, resulting in increased tear film production.^[5]^ Several randomized controlled trials (RCTs) have shown that Tyrvaya is effective in alleviating symptoms of DED, including improvements in Schirmer’s Test Score and overall dry eye symptom scores.^[8–10]^ The intranasal route of administration (ROA) is known to cause nasal cavity side effects, the use of an indirect ROA to treat ocular diseases raises concerns about the safety of this unique intervention.^[11]^ Most reported adverse events (AEs) associated with Tyrvaya are mild, including transient sneezing and coughing, and it is generally regarded as having a favorable safety profile,^[12]^ however, a comprehensive evaluation of its long-term safety in clinical practice is lacking.

FAERS is one of the largest post-market safety monitoring databases. It collects standardized real-world data to support the FDA’s safety surveillance program for drugs and therapeutic biologics through spontaneous reports from consumers, healthcare professionals, pharmaceutical manufacturers, and other nonmedical individuals.^[13]^ In this study, we performed a comprehensive analysis of AEs associated with Tyrvaya in the FAERS database using 4 algorithms: reporting odds ratio (ROR), proportional reporting ratio (PRR), multi-item gamma Poisson shrinker (MGPS), and Bayesian confidence propagation neural network (BCPNN). In addition to validating known AEs, this study would investigate unreported and potential AEs not described on the drug label, with the goal of providing new safety-related insights for clinical ophthalmologists using Tyrvaya to treat DED.

## 2. Materials and methods

### 2.1. Data source and study design

**Figure 1.**
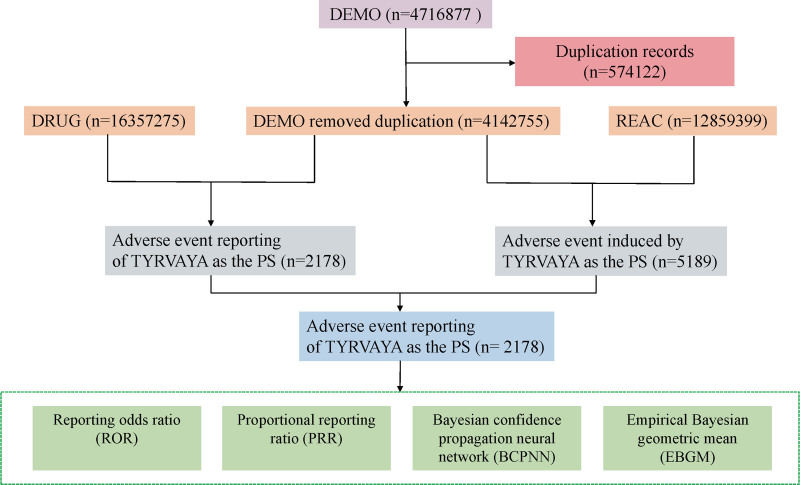
Flowchart of the screening process for Tyrvaya-related adverse events. BCPNN = Bayesian confidence propagation neural network, DEMO = comprising patient demographic and administrative details, EBGM = empirical Bayesian geometric mean. PS = primary suspect, ROR = reporting odds ratio, PRR = proportional reporting ratio

The data for this study were obtained from the FAERS database, a large-scale pharmacovigilance resource that supports the FDA’s post-marketing surveillance program for all approved drugs and therapeutic biologics. The FAERS dataset primarily consists of 7 different sections, including DEMO (comprising patient demographic and administrative details), DRUG (housing drug-specific information), REAC (coded representations of reported AEs), OUTC (reflecting patient outcomes), RPSR (indicating sources of reports), THER (documenting therapy initiation and cessation dates for reported drugs), and INDI (outlining indications for drug administration). The FAERS categorizes reported drugs into 4 categories: PS (primary suspect), SS (secondary suspect), C (concomitant), and I (interacting). In this study, we focused exclusively on data that designated Tyrvaya as a PS. AEs and medication errors are coded using terminology from the Medical Dictionary for Regulatory Activities (MedDRA), a comprehensive and detailed standard developed by the International Council for Harmonisation of Technical Requirements for Pharmaceuticals for Human Use (ICH). To address duplicate reports, we adopted the methodology recommended by the FDA. From the DEMO table, we extracted the PRIMARYID, CASEID, and FDA_DT fields, selecting entries with the maximum FDA_DT value according to FDA guidelines to ensure that we retained the most recent report for each CASEID. In cases where CASEID and FDA_DT are identical, the report with the highest PRIMARYID was retained. The detailed flowchart of the research design can be found in Figure 1.

### 2.2. Statistical analysis

Descriptive analysis was utilized to characterize the features of AEs reports associated with Tyrvaya. Descriptive analysis typically comprises 2 components: Frequentist Statistics and Bayesian Statistics. Frequentist Statistics includes the ROR^[14]^ and PRR,^[15]^ while Bayesian Statistics encompasses the MGPS^[16]^ and BCPNN.^[17]^ In this study, AEs that simultaneously meet the positive thresholds of all 4 methods were classified as adverse reactions, whereas those that meet the positive thresholds of 2 or 3 methods were also deemed noteworthy. The interval between the occurrence of AEs recorded in the DEMO file and the initiation of Tyrvaya treatment documented in the THER file is defined as the onset time of Tyrvaya-related AEs. The Weibull distribution was utilized to model temporal changes in the incidence of AEs. All analyses were conducted using R software version 4.3.0. The 2 × 2 contingency table underlying the descriptive analysis can be found in Table S1, Supplemental Digital Content, https://links.lww.com/MD/Q253, while the specific algorithms and positive threshold criteria for the 4 methods are outlined in Table S2, Supplemental Digital Content, https://links.lww.com/MD/Q253.

## 3. Results

### 3.1. General characteristics

From the fourth quarter of 2021 to the second quarter of 2024, a total of 5189 AEs are expected to be reported among 2178 patients for whom Tyrvaya was regarded as the primary suspected drug. The proportion of reported female patients surpassed that of male patients, representing 74% and 11.8%, respectively. Regarding age, there was a substantial amount of unknown data; however, among the available data, the largest proportion of patients fell within the 65 to 85 age range, accounting for approximately 16.9%. The United States was the only reporting country, accounting for 100%. In terms of reporter, consumers constituted the largest proportion at 85.1%, followed by health professionals, physicians, and pharmacists. The number of reported AEs had demonstrated a consistent yearly increase, reaching the highest proportion of 48.71% in 2024, followed by 27.36% in 2023 and 23.78% in 2022. The basic information is provided in Table 1.

**Table 1:** Clinical characteristics of Tyrvaya adverse event reports from the FAERS database (Q4 2021–Q2 2024).

Characteristics	Case numbers	Case proportion (%)
Number of events	2178	
Gender		
Male	257	11.8
Female	1611	74.0
Miss	310	14.2
Age		
18–65	227	10.4
65–85	368	16.9
>85	32	1.5
Miss	1551	71.2
Reporting countries		
United States	2178	100
Reporter		
Consumer	1854	85.1
Health professionals	131	6.0
Physician	187	8.6
Pharmacist	4	0.2
Miss	2	0.1
Reporting year		
2021	3	0.14
2022	518	23.78
2023	596	27.36
2024	1061	48.71

FAERS = The FDA Adverse Event Reporting System.

### 3.2. Signal detection of Tyrvaya at the system organ class (SOC) level

**Figure 2.**
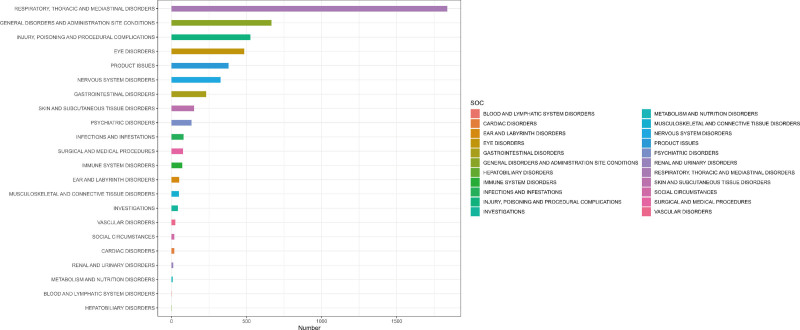
Proportion of adverse events by system organ class for Tyrvaya. SOC = system organ class.

When Tyrvaya was considered the PS drug, the AEs associated with it involve 22 organ systems. (The specific details can be found in Table 2, The proportion of SOC reported in Tyrvaya-related AEs can be found in Fig. 2.) Regarding the number of reports, the most commonly reported system was respiratory, thoracic, and mediastinal disorders (n = 1837), followed by 4 additional prevalent systems: general disorders and administration site conditions (n = 666), injury, poisoning and procedural complications (n = 525), eye disorders (n = 484), and product issues (n = 380). In the context of signal strength detection, the systems that concurrently met the positive thresholds of the 4 algorithms include respiratory, thoracic, and mediastinal disorders (ROR = 11.72, PRR = 7.93, EBGM = 7.91, IC = 2.98), eye disorders (ROR = 5.17, PRR = 4.78, EBGM = 4.78, IC = 2.26), and product issues (ROR = 3.88, PRR = 3.67, EBGM = 3.66, IC = 1.87). It is worth noting that eye disorders, as a new SOC signal, was not mentioned in previous product labeling for Tyrvaya. Additionally, we found that Ear and Labyrinth Disorders attained the positive thresholds of 2 algorithms concurrently (ROR = 2.51, PRR = 2.49).

**Table 2:** Signal strength of Tyrvaya adverse events across system organ classes (SOC) in the FAERS database.

System organ class (SOC)	Case numbers	ROR (95% CI)	PRR (χ^2^)	EBGM (EBGM05)	IC (IC025)
Skin and subcutaneous tissue disorders	150	0.56 (0.47–0.66)	0.57 (50.78)	0.57 (0.5)	−0.81 (−2.47)
General disorders and administration site conditions	666	0.67 (0.62–0.73)	0.72 (91.82)	0.72 (0.67)	−0.48 (−2.15)
Respiratory, thoracic and mediastinal disorders*	1837	11.72 (11.08–12.41)	7.93 (11,605.08)	7.91 (7.54)	2.98 (1.32)
Immune system disorders	71	1.24 (0.98–1.57)	1.24 (3.27)	1.24 (1.02)	0.31 (−1.36)
Nervous system disorders	326	0.87 (0.78–0.98)	0.88 (5.69)	0.88 (0.8)	−0.18 (−1.85)
Gastrointestinal disorders	231	0.55 (0.48–0.63)	0.57 (81.5)	0.57 (0.51)	−0.81 (−2.48)
Psychiatric disorders	133	0.47 (0.39–0.56)	0.48 (78.71)	0.48 (0.42)	−1.06 (−2.72)
Renal and urinary disorders	11	0.12 (0.07–0.23)	0.13 (67.48)	0.13 (0.08)	−2.98 (−4.65)
Cardiac disorders	18	0.18 (0.12–0.29)	0.19 (65.3)	0.19 (0.13)	−2.43 (−4.09)
Investigations	42	0.13 (0.1–0.18)	0.14 (240.71)	0.14 (0.11)	−2.86 (−4.53)
Injury, poisoning and procedural complications	525	0.74 (0.68–0.81)	0.77 (41.91)	0.77 (0.71)	−0.38 (−2.05)
Product issues*	380	3.88 (3.49–4.31)	3.67 (751.43)	3.66 (3.36)	1.87 (0.21)
Eye disorders*	484	5.17 (4.71–5.68)	4.78 (1473.93)	4.78 (4.42)	2.26 (0.59)
Infections and infestations	80	0.25 (0.2–0.31)	0.26 (181.52)	0.26 (0.21)	−1.95 (−3.62)
Vascular disorders	25	0.26 (0.18–0.39)	0.26 (51.96)	0.26 (0.19)	−1.92 (−3.58)
Ear and labyrinth disorders	52	2.51 (1.91–3.29)	2.49 (46.55)	2.49 (1.98)	1.32 (-0.35)
Musculoskeletal and connective tissue disorders	50	0.18 (0.13–0.23)	0.18 (189.82)	0.18 (0.15)	−2.44 (−4.1)
Social circumstances	19	0.8 (0.51–1.26)	0.8 (0.91)	0.8 (0.55)	−0.31 (−1.98)
Metabolism and nutrition disorders	8	0.08 (0.04–0.16)	0.08 (84.07)	0.08 (0.05)	−3.61 (−5.28)
Hepatobiliary disorders	1	0.02 (0–0.16)	0.02 (41.38)	0.02 (0)	−5.43 (−7.09)
Surgical and medical procedures	77	0.94 (0.75–1.18)	0.94 (0.3)	0.94 (0.78)	−0.09 (−1.76)
Blood and lymphatic system disorders	2	0.02 (0.01–0.09)	0.02 (88.84)	0.02 (0.01)	−5.51 (−7.18)

AEs = adverse events, CI = confidence interval, EBGM = empirical Bayesian geometric mean, EBGM05 = the lower limit of the 95% CI of EBGM, FAERS = The FDA Adverse Event Reporting System, IC = information component, IC025 = the lower limit of the 95% CI of the IC, PRR = proportional reporting ratio, ROR = reporting odds ratio, SOC = system organ class.

* Statistically significant signals in 4 algorithms.

### 3.3. Signal detection of Tyrvaya at the preferred terms (PT) level

**Figure 3.**
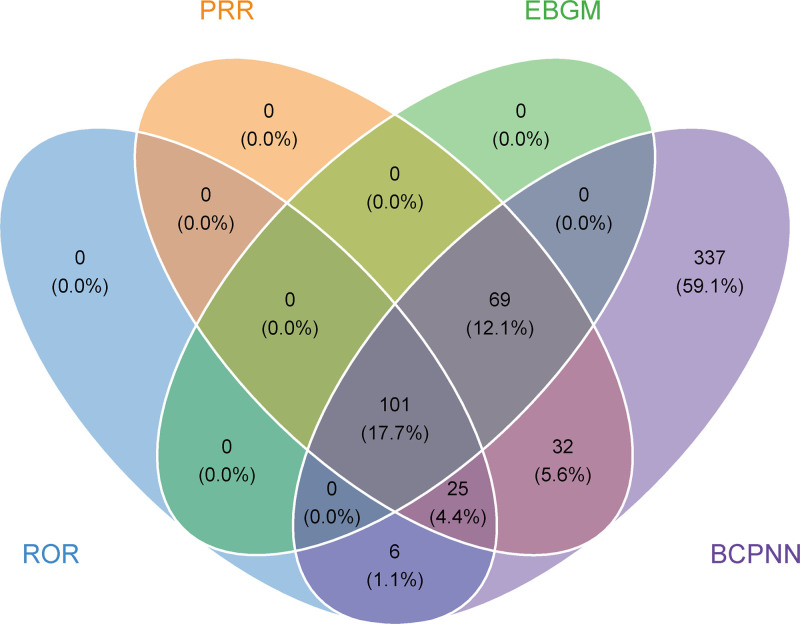
Venn diagram of preferred term (PT) signals meeting the criteria of 4 algorithms. BCPNN = Bayesian confidence propagation neural network, EBGM = empirical Bayesian geometric mean, PRR = proportional reporting ratio. PT = preferred term, ROR = reporting odds ratio.

At the preferred term (PT) level, we organized the AEs associated with Tyrvaya according to their frequency and analyzed the positive signals using 4 distinct algorithms. The Venn diagram in Figure 3 visually illustrated the AEs that met the positive threshold of all 4 algorithms at the PT level. Among the 50 most prevalent AEs, reactions explicitly listed in the product labeling were identified, including sneezing, throat irritation, and cough. In addition, we also identified AEs that were not mentioned in the product labeling, including nasal discomfort, rhinorrhea, epistaxis, eye irritation, oropharyngeal pain, increased lacrimation, eye pain, rhinalgia, ocular hyperemia, nasal congestion, blurred vision, burning sensation, upper-airway cough syndrome, eye pruritus, nasal dryness, nasal ulcer, dysphonia, abnormal dreams, throat tightness, photophobia, aftertaste, and eye swelling. At the same time, we also identified several AEs that simultaneously met the positive thresholds of 2 or 3 algorithms, including headache, insomnia, hypersensitivity, sinusitis, dry mouth, and vertigo, which were similarly noteworthy. Detailed information on the above content can be found in Table 3.

**Table 3:** Top 50 frequency of adverse events at the PT level for Tyrvaya.

PT	Case numbers	ROR (95% CI)	PRR (χ^2^)	EBGM (EBGM05)	IC (IC025)
Sneezing*	603	353.98 (323.67–387.13)	312.96 (1,66,547.77)	277.97 (257.91)	8.12 (6.45)
Nasal discomfort*	213	498.81 (429.63–579.13)	478.37 (85,053.44)	401.11 (354)	8.65 (6.98)
Rhinorrhoea*	179	30.29 (26.08–35.2)	29.28 (4838.6)	28.95 (25.54)	4.86 (3.19)
Throat irritation*	150	41.98 (35.64–49.44)	40.79 (5732.38)	40.15 (35.01)	5.33 (3.66)
Epistaxis*	133	27.89 (23.46–33.16)	27.2 (3323.72)	26.92 (23.29)	4.75 (3.08)
Cough*	127	4.95 (4.15–5.9)	4.85 (389.21)	4.84 (4.18)	2.28 (0.61)
Headache	119	2.58 (2.15–3.09)	2.54 (112.15)	2.54 (2.18)	1.34 (-0.32)
Eye irritation*	68	16.26 (12.79–20.68)	16.06 (955.2)	15.97 (13.06)	4 (2.33)
Oropharyngeal pain*	64	7.55 (5.9–9.66)	7.47 (358.09)	7.45 (6.06)	2.9 (1.23)
Lacrimation increased*	57	21.79 (16.76–28.32)	21.56 (1108.53)	21.38 (17.17)	4.42 (2.75)
Eye pain*	44	9.97 (7.4–13.42)	9.89 (350.63)	9.86 (7.69)	3.3 (1.63)
Hypersensitivity	43	3.04 (2.25–4.11)	3.02 (58.36)	3.02 (2.35)	1.6 (−0.07)
Rhinalgia*	42	158.92 (116.2–217.35)	157.64 (6146.38)	148.27 (114.1)	7.21 (5.54)
Ocular hyperemia*	41	10.63 (7.81–14.46)	10.55 (353.29)	10.51 (8.12)	3.39 (1.73)
Insomnia	39	2.22 (1.62–3.04)	2.21 (25.78)	2.21 (1.69)	1.14 (−0.53)
Dyspnoea	36	0.85 (0.61–1.18)	0.85 (0.94)	0.85 (0.65)	−0.23 (−1.9)
Nasal congestion*	36	6.21 (4.47–8.62)	6.17 (155.86)	6.16 (4.68)	2.62 (0.96)
Vision blurred*	36	3.71 (2.67–5.15)	3.69 (70.75)	3.69 (2.8)	1.88 (0.22)
Burning sensation*	35	7.29 (5.22–10.17)	7.25 (188.04)	7.23 (5.47)	2.85 (1.19)
Dizziness	34	0.95 (0.68–1.33)	0.95 (0.1)	0.95 (0.71)	−0.08 (−1.74)
Rash	33	0.92 (0.65–1.3)	0.92 (0.22)	0.92 (0.69)	−0.12 (−1.79)
Nausea	29	0.5 (0.34–0.72)	0.5 (14.7)	0.5 (0.37)	−1 (−2.67)
Feeling abnormal	25	1.49 (1.01–2.21)	1.49 (4.02)	1.49 (1.07)	0.57 (−1.09)
Pain	25	0.37 (0.25–0.55)	0.37 (26.63)	0.37 (0.27)	−1.42 (−3.09)
Anxiety	21	1 (0.65–1.53)	1 (0)	1 (0.7)	0 (−1.67)
Upper-airway cough syndrome*	20	25.38 (16.33–39.47)	25.29 (461.96)	25.05 (17.31)	4.65 (2.98)
Erythema	20	1.44 (0.93–2.23)	1.44 (2.66)	1.44 (0.99)	0.52 (−1.14)
Eye pruritus*	20	6.18 (3.98–9.6)	6.16 (86.33)	6.15 (4.26)	2.62 (0.95)
Nasal dryness*	19	41.43 (26.31–65.26)	41.29 (734.73)	40.63 (27.78)	5.34 (3.68)
Sinusitis	19	2.03 (1.29–3.18)	2.02 (9.86)	2.02 (1.39)	1.02 (−0.65)
Pruritus	18	0.55 (0.34–0.87)	0.55 (6.8)	0.55 (0.37)	−0.87 (−2.54)
Drug hypersensitivity	18	1.36 (0.86–2.16)	1.36 (1.71)	1.36 (0.92)	0.44 (−1.22)
Nasal ulcer*	17	152.53 (93.4–249.1)	152.03 (2403.2)	143.3 (95.06)	7.16 (5.49)
Urticaria	16	1.32 (0.81–2.16)	1.32 (1.26)	1.32 (0.88)	0.4 (−1.26)
Palpitations	16	1.99 (1.22–3.25)	1.99 (7.84)	1.99 (1.32)	0.99 (−0.68)
Visual impairment	16	1.5 (0.92–2.45)	1.5 (2.63)	1.49 (0.99)	0.58 (−1.09)
Dysphonia*	16	3.32 (2.03–5.42)	3.31 (25.8)	3.31 (2.19)	1.73 (0.06)
Abnormal dreams*	15	13.32 (8.01–22.13)	13.28 (169.46)	13.21 (8.64)	3.72 (2.06)
Migraine	14	1.73 (1.02–2.92)	1.72 (4.26)	1.72 (1.11)	0.79 (−0.88)
Throat tightness*	14	7.56 (4.47–12.79)	7.54 (79.26)	7.52 (4.85)	2.91 (1.24)
Photophobia*	14	9.26 (5.48–15.66)	9.24 (102.5)	9.21 (5.93)	3.2 (1.54)
Product after taste*	13	59.67 (34.4–103.51)	59.53 (730.54)	58.15 (36.68)	5.86 (4.19)
Eye swelling*	13	5.18 (3–8.93)	5.17 (43.66)	5.16 (3.27)	2.37 (0.7)
Fatigue	13	0.19 (0.11–0.32)	0.19 (46.28)	0.19 (0.12)	−2.41 (−4.08)
Dry mouth	13	2.29 (1.33–3.95)	2.29 (9.42)	2.29 (1.45)	1.19 (−0.47)
Nasopharyngitis	12	0.68 (0.39–1.21)	0.68 (1.75)	0.68 (0.43)	−0.55 (−2.21)
Vertigo	12	3.02 (1.71–5.32)	3.01 (16.15)	3.01 (1.87)	1.59 (−0.08)
Vomiting	12	0.36 (0.2–0.63)	0.36 (13.76)	0.36 (0.22)	−1.47 (−3.14)
Heart rate increased	12	1.51 (0.86–2.66)	1.51 (2.05)	1.51 (0.94)	0.59 (−1.07)
Abdominal discomfort	11	0.73 (0.4–1.31)	0.73 (1.12)	0.73 (0.44)	−0.46 (−2.12)

CI = confidence interval, EBGM = empirical Bayesian geometric mean, EBGM05 = the lower limit of the 95% CI of EBGM, IC = information component, IC025 = the lower limit of the 95% CI of the IC, PRR = proportional reporting ratio, PT = preferred term, ROR = reporting odds ratio.

* Statistically significant signals in 4 algorithms.

### 3.4. Time-to-onset analysis and weibull distribution analysis

**Figure 4.**
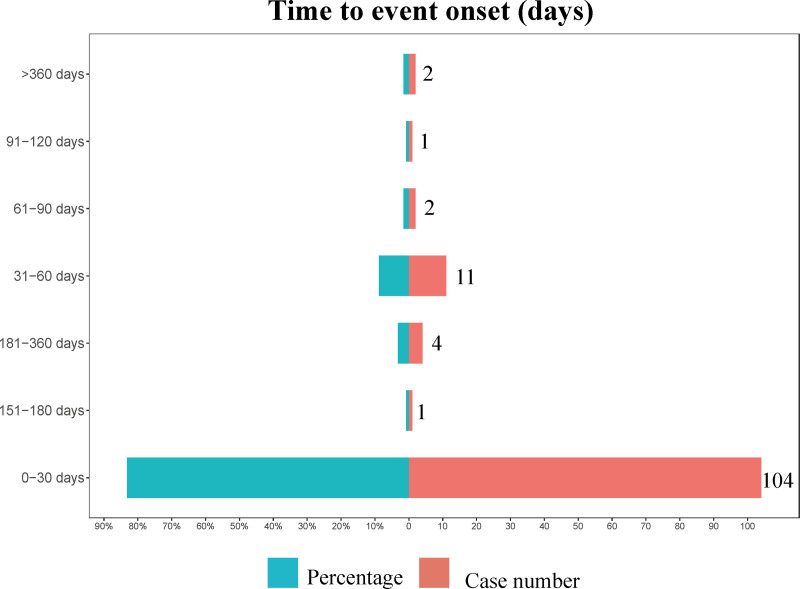
Time to onset of Tyrvaya-related adverse events.

**Figure 5.**
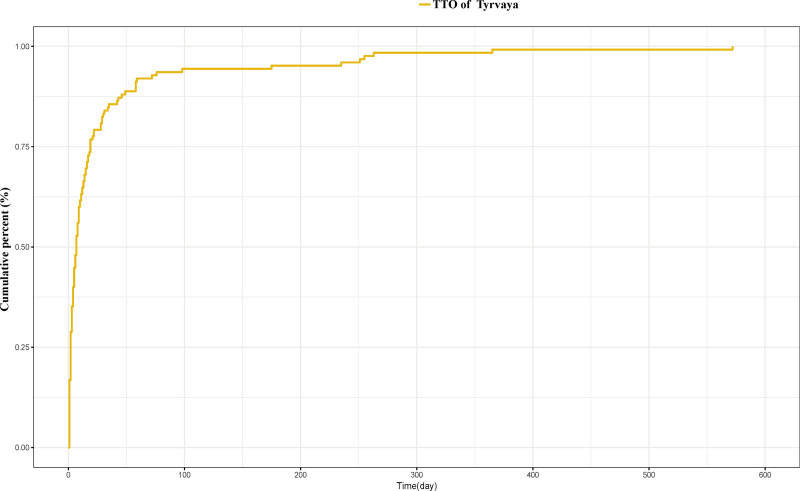
Cumulative incidence of adverse events related to Tyrvaya over time. TTO = time to onset.

A total of 125 AEs were associated with onset time, predominantly occurring within the first month. The average onset time was 29.3 days, with a median of 7 days (interquartile range [IQR]: 2–19 days). The distribution of the onset times for the above AEs can be found in Figure 4. Analysis utilizing the Weibull distribution revealed an early failure mode. The detailed parameters of this analysis are presented in Table 4. Furthermore, the cumulative incidence curve of AEs is depicted in Figure 5.

**Table 4:** Time to onset of Tyrvaya-associated adverse events and Weibull distribution analysis.

Drug	TTO (d)		Weibull distribution		
	Case reports	Median (d; IQR)	Scale parameter: α (95% CI)	Shape parameter: β (95% CI)	Type
Tyrvaya	125	7 (2–19)	16.73 (11.53–21.92)	0.60 (0.52–0.67)	Early failure

CI = confidence interval, IQR = interquartile range, TTO = time to onset.

## 4. Discussion

This study represented the first application of the FAERS database to evaluate the real-world safety of Tyrvaya since its launch in 2021. We utilized 4 algorithms – ROR, PRR, MGPS, and BCPNN – to evaluate the AEs associated with Tyrvaya as the principal suspected drug. The ROR calculation method exhibits high sensitivity and simplicity in calculation, however, it is susceptible to producing false positive signals when the volume of reports is limited. PRR is a straightforward disproportionality measure designed to identify potential signals of adverse drug reactions. BCPNN and MGPS demonstrate high specificity.^[13]^ BCPNN employs Bayesian methods to estimate the probability of a causal relationship between a drug and an adverse event, whereas MGPS adjusts effect sizes (such as PRR or ROR) to minimize the incidence of false positive signals.^[18]^ Each of these algorithms has its own advantages and limitations. Consequently, this study focused on discussing AEs that conform to the criteria of 2 or more algorithms, particularly those that satisfy all 4.

This research confirmed the AEs already found on the Tyrvaya drug label, including sneezing, throat irritation, and cough, and verified that sneezing is the most common adverse reaction. In addition, we discovered AEs not mentioned in the drug label, with those related to eye disorders being the most common. These include eye irritation, increased lacrimation, eye pain, ocular hyperemia, blurred vision, eye itching, photophobia, and eye swelling. AEs related to respiratory, thoracic and mediastinal disorders, as well as psychiatric disorders, were also discovered. Our findings indicated that reports from female patients substantially outnumber those from male patients, with the United States as the sole reporting country in this study. Additionally, there has been a yearly increase in the number of reported incidents, peaking in the first half of 2024. AEs associated with Tyrvaya primarily occurred around the first month of treatment, accounting for approximately 83.2%.

Tyrvaya is the commercial name for the varenicline nasal spray, which is a small molecule nAChR agonist.^[19]^ Upon administration, the varenicline molecule interacts with nAChRs on the free nerve endings in the nasociliary and maxillary branches of the trigeminal nerve in the nasal mucosa, this interaction initiates the depolarization of the nerve that innervates the lacrimal functional unit, thereby enhancing tear film production through the activation of ligand-gated ion channels.^[12]^ Tyrvaya is free from preservatives and does not directly contact the eyes, thereby minimizing the risk of toxic conjunctivitis. Additionally, its route of administration is more convenient, enhancing suitability for contact lens users.^[4]^ Varenicline is a widely used smoking cessation medication, but Tyrvaya administers it via nasal spray, resulting in extremely low absorption in the blood, and each dose contains only 0.03mg of varenicline.^[20]^ Considering the benefits and growing prevalence of Tyrvaya in treating DED, assessing its safety profile is crucial.

Following a disproportionality analysis of the FAERS database, predominant signals at the SOC level linked to Tyrvaya predominantly encompassed respiratory, thoracic, and mediastinal disorders, product issues, and eye disorders. Among these, certain AEs such as sneezing, throat irritation, and cough, identified within the category of respiratory, thoracic, and mediastinal disorders, aligned with the descriptions found on the Tyrvaya drug label. Two RCTs examining Tyrvaya treatment for DED identified associated AEs, with sneezing being the most prevalent.^[21,22]^ Additionally, a meta-analysis conducted by Bashrahil B et al^[5]^ revealed that sneezing was the predominant nasal-related AE, succeeded by coughing and throat irritation. Additionally, we identified some AEs within the respiratory, thoracic, and mediastinal disorders categories that were not reported in the instructions, including epistaxis, oropharyngeal pain, rhinalgia, nasal congestion, upper-airway cough syndrome, nasal dryness, nasal ulcer, dysphonia, and throat tightness. Tyrvaya is a nasal spray that can cause reflex responses, mediated by triggering the trigeminal parasympathetic pathway in the nasal cavity.^[8]^ This mechanical method of administration may help explain the occurrence of the aforementioned adverse reactions.

Tyrvaya does not directly interact with the ocular surface, and reported cases of eye-related AEs are relatively rare.^[23]^ Additionally, the drug’s labeling did not explicitly reference any ocular adverse reactions. Notably, this study identified “Eye disorders” as one of the signals that met the positive threshold across all 4 algorithms at the SOC level. Among the top 50 PT signals by frequency, the corresponding AEs, including eye irritation, lacrimation increased, eye pain, ocular hyperemia, vision blurred, eye pruritus, photophobia, and eye swelling, also met the positive threshold across all 4 algorithms. A study by Hauswirth SG et al^[24]^ reported that eye-related AEs comprised 12.7% to 12.9% of cases involving varenicline nasal spray, with the most frequently reported symptoms being reduced visual acuity (3.6–3.7%) and conjunctival hyperemia (3.3–3.7%). A clinical trial by Wirta D et al^[9]^ reported 1 case of eyelid edema as an adverse reaction in a patient using a spray containing 0.06 mg of varenicline per dose. Quiroz-Mercado H et al^[21]^ reported 9 cases of eye-related AEs in a study of 97 patients with DED, among these, 7 cases involved vision impairment: 4 patients used a spray containing 0.03 mg of varenicline per dose, while 3 patients used a spray containing 0.06 mg per dose. A meta-analysis by Ballesteros-Sánchez A et al^[12]^ found that the most frequently reported eye-related adverse event associated with varenicline nasal spray was reduced visual acuity. The mechanism underlying eye-related AEs associated with Tyrvaya remains unclear. However, given the findings of this study, the issue warrants further attention. Clinicians should remain vigilant for eye-related AEs when prescribing Tyrvaya for the treatment of DED.

This study identified several previously unreported AEs, including abnormal dreams, which met the positive threshold across all 4 algorithms. Furthermore, certain AEs also warrant attention, such as headache, insomnia, hypersensitivity, sinusitis, dry mouth, and vertigo, as they met the positive threshold in 2 or 3 algorithms. A study reported that 1 patient receiving varenicline nasal spray withdrew on the first day of treatment due to a headache, while another patient withdrew on the thirteenth day after developing a headache.^[25]^ Interestingly, in this research, headache was identified as an adverse event that met the positive threshold in 3 algorithms, highlighting the need for clinicians to monitor this type of reaction closely. An analysis of the timing of Tyrvaya-related AEs revealed that the majority of cases occurred within the first month of treatment. These findings highlight the importance of clinicians maintaining heightened vigilance for adverse reactions during this initial treatment period.

This study offered several strengths. First, we employed 4 distinct disproportionality analysis algorithms to assess AEs data related to Tyrvaya. By integrating the advantages of these algorithms, we aimed to enhance the reliability and stability of the findings. Secondly, the FAERS database is based on real-world data, enabling the collection of large sample sizes. These data can originate from a wide range of sources, such as consumers, doctors, nurses, or other healthcare professionals. This study had several limitations. First, the majority of reports were submitted by consumers, with relatively few submissions from professional doctors or other medical personnel. Secondly, all the data in this study were reported from a single country, raising the question of whether differences exist across various ethnicities or economic levels. Thirdly, while data mining techniques helped identify adverse reaction signals associated with Tyrvaya, this method alone does not establish a causal relationship. Large-scale, high-quality prospective studies are still needed in the future to further investigate AEs related to Tyrvaya.

## 5. Conclusion

This study analyzed and discussed AEs associated with Tyrvaya, utilizing data from the FAERS database over the period from its market introduction in 2021 to the second quarter of 2024. In addition to confirming the known adverse reactions explicitly listed in the drug label, this study also identified potential adverse reactions not previously mentioned. Adverse reactions related to eye disorders should receive particular attention, and the adverse event of headache also warrants careful consideration. In conclusion, this study offered valuable insights into the post-marketing safety of Tyrvaya.

## Author contributions

**Conceptualization:** Chang-Zhu He, Yu He.

**Data curation:** Chang-Zhu He, Wei-Yu Wang.

**Formal analysis:** Chang-Zhu He, Qin Qiu, Yu He.

**Methodology:** Chang-Zhu He, Yu He.

**Software:** Chang-Zhu He.

**Writing – original draft:** Chang-Zhu He, Song-Jie Lu, Fu-Li Xue.

**Writing – review & editing:** Chang-Zhu He, Yu He.

## Supplementary Material



## References

[1] Craig JP Nichols KK Akpek EK. TFOS DEWS II definition and classification report. Ocul Surf. 2017;15:276–83. 28736335 10.1016/j.jtos.2017.05.008

[2] Deo N Nagrale P. Dry eye disease: an overview of its risk factors, diagnosis, and prevalence by age, sex, and race. Cureus. 2024;16:e54028. 38481927 10.7759/cureus.54028 PMC10934010

[3] Papas EB. The global prevalence of dry eye disease: a Bayesian view. Ophthalmic Physiol Opt. 2021;41:1254–66. 34545606 10.1111/opo.12888

[4] Wong JC Barak A. Managing dry eye disease with novel medications: mechanism, study validity, safety, efficacy, and practical application. Pharmacy (Basel). 2024;12:19. 38392926 10.3390/pharmacy12010019 PMC10892551

[5] Bashrahil B Taher N Alzahrani Z. The efficacy and safety of varenicline nasal spray for the management of dry eye signs: a systematic review and meta-analysis. BMC Ophthalmol. 2023;23:319. 37452334 10.1186/s12886-023-03069-y PMC10347795

[6] Mihalak KB Carroll FI Luetje CW. Varenicline is a partial agonist at alpha4beta2 and a full agonist at alpha7 neuronal nicotinic receptors. Mol Pharmacol. 2006;70:801–5. 16766716 10.1124/mol.106.025130

[7] Bordia T Hrachova M Chin M McIntosh JM Quik M. Varenicline is a potent partial agonist at α6β2* nicotinic acetylcholine receptors in rat and monkey striatum. J Pharmacol Exp Ther. 2012;342:327–34. 22550286 10.1124/jpet.112.194852 PMC3400806

[8] Tian L Jin X Wang J. Varenicline solution nasal spray for dry eye disease in Chinese patients: a randomized phase 3 trial. Lancet Reg Health West Pac. 2024;45:101032. 38440130 10.1016/j.lanwpc.2024.101032 PMC10909742

[9] Wirta D Vollmer P Paauw J. Efficacy and safety of OC-01 (varenicline solution) nasal spray on signs and symptoms of dry eye disease: the ONSET-2 phase 3 randomized trial. Ophthalmology. 2022;129:379–87. 34767866 10.1016/j.ophtha.2021.11.004

[10] Katz J Periman LM Maiti S. Bilateral effect of OC-01 (varenicline solution) nasal spray for treatment of signs and symptoms in individuals with mild, moderate, and severe dry eye disease. Clin Ther. 2022;44:1463–70. 36763994 10.1016/j.clinthera.2022.09.013

[11] Pflugfelder SC Cao A Galor A Nichols KK Cohen NA Dalton M. Nicotinic acetylcholine receptor stimulation: a new approach for stimulating tear secretion in dry eye disease. Ocul Surf. 2022;25:58–64. 35550851 10.1016/j.jtos.2022.05.001

[12] Ballesteros-Sánchez A Borroni D De-Hita-Cantalejo C. Efficacy of bilateral OC-01 (varenicline solution) nasal spray in alleviating signs and symptoms of dry eye disease: a systematic review. Cont Lens Anterior Eye. 2024;47:102097. 38065797 10.1016/j.clae.2023.102097

[13] Zhao J Tao Y. Adverse event reporting of the IGF-1R monoclonal antibody teprotumumab: a real-world study based on the US food and drug administration adverse event reporting system. Front Pharmacol. 2024;15:1393940. 39185318 10.3389/fphar.2024.1393940 PMC11341477

[14] Rothman KJ Lanes S Sacks ST. The reporting odds ratio and its advantages over the proportional reporting ratio. Pharmacoepidemiol Drug Saf. 2004;13:519–23. 15317031 10.1002/pds.1001

[15] Evans SJ Waller PC Davis S. Use of proportional reporting ratios (PRRs) for signal generation from spontaneous adverse drug reaction reports. Pharmacoepidemiol Drug Saf. 2001;10:483–6. 11828828 10.1002/pds.677

[16] Bate A Lindquist M Edwards IR. A Bayesian neural network method for adverse drug reaction signal generation. Eur J Clin Pharmacol. 1998;54:315–21. 9696956 10.1007/s002280050466

[17] Szarfman A Tonning JM Doraiswamy PM. Pharmacovigilance in the 21st century: new systematic tools for an old problem. Pharmacotherapy. 2004;24:1099–104. 15460169 10.1592/phco.24.13.1099.38090

[18] Wang K Wang M Li W Wang X. A real-world disproportionality analysis of Tivozanib data mining of the public version of FDA adverse event reporting system. Front Pharmacol. 2024;15:1408135. 38939844 10.3389/fphar.2024.1408135 PMC11208458

[19] Cui D Saldanha IJ Li G Mathews PM Lin MX Akpek EK. United States regulatory approval of topical treatments for dry eye. Am J Ophthalmol. 2024;258:14–21. 37793479 10.1016/j.ajo.2023.09.024

[20] Zitko KL Ladd L Dougherty TS. Intranasal varenicline: review of a novel formulation for the treatment of dry eye disease. J Pharm Pract. 2023;36:1448–53. 35703427 10.1177/08971900221108725

[21] Quiroz-Mercado H Hernandez-Quintela E Chiu KH Henry E Nau JA. A phase II randomized trial to evaluate the long-term (12-week) efficacy and safety of OC-01 (varenicline solution) nasal spray for dry eye disease: the MYSTIC study. Ocul Surf. 2022;24:15–21. 34920097 10.1016/j.jtos.2021.12.007

[22] Wirta D Torkildsen GL Boehmer B. ONSET-1 phase 2b randomized trial to evaluate the safety and efficacy of OC-01 (varenicline solution) nasal spray on signs and symptoms of dry eye disease. Cornea. 2022;41:1207–16. 36107843 10.1097/ICO.0000000000002941 PMC9473713

[23] Torkildsen GL Pattar GR Jerkins G Striffler K Nau J. Efficacy and safety of single-dose OC-02 (simpinicline solution) nasal spray on signs and symptoms of dry eye disease: the PEARL phase II randomized trial. Clin Ther. 2022;44:1178–86. 35965109 10.1016/j.clinthera.2022.07.006

[24] Hauswirth SG Kabat AG Hemphill M Somaiya K Hendrix LH Gibson AA. Safety, adherence and discontinuation in varenicline solution nasal spray clinical trials for dry eye disease. J Comp Eff Res. 2023;12:e220215. 37096956 10.57264/cer-2022-0215 PMC10402908

[25] Frampton JE. Varenicline solution nasal spray: a review in dry eye disease. Drugs. 2022;82:1481–8. 36197638 10.1007/s40265-022-01782-4 PMC9533262

